# 1-Furfuryl-3-furoylthio­urea

**DOI:** 10.1107/S1600536808015250

**Published:** 2008-05-24

**Authors:** O. Estévez-Hernández, J. Duque, J. Ellena, Rodrigo S. Corrêa

**Affiliations:** a Department of Structure Analysis, Institute of Materials, University of Havana, Cuba; b Grupo de Cristalografía, Instituto de Física de São Carlos, Universidade de São Paulo, São Carlos, Brazil

## Abstract

The title compound, C_11_H_10_N_2_O_3_S, was synthesized from furoyl isothio­cyanate and furfurylamine in dry acetone. The thio­urea group is in the thio­amide form. The *trans*–*cis* geometry of the thio­urea group is stabilized by intra­molecular hydrogen bonding between the carbonyl and *cis*-thio­amide and results in a pseudo-*S*(6) planar ring which makes dihedral angles of 2.5 (3) and 88.1 (2)° with the furoyl and furfuryl groups, respectively. There is also an intra­molecular hydrogen bond between the furan O atom and the other thio­amide H atom. In the crystal structure, mol­ecules are linked by two inter­molecular N—H⋯O hydrogen bonds, forming dimers. These dimers are stacked within the crystal structure along the [010] direction.

## Related literature

For general background, see: Dhooghe *et al.* (2005 ▶); Aly *et al.* (2007 ▶); Estévez-Hernández *et al.* (2007 ▶). For related structures, see: Koch (2001 ▶); Yamin & Hassan (2004 ▶). For the synthesis, see: Otazo *et al.* (2001 ▶).
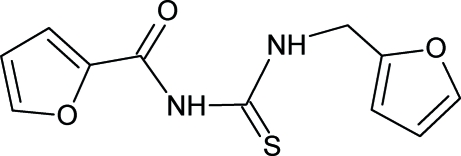


## Experimental

### 

#### Crystal data

C_11_H_10_N_2_O_3_S
*M* *_r_* = 250.27
Triclinic, 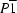

*a* = 4.5999 (2) Å
*b* = 11.3792 (6) Å
*c* = 12.0556 (5) Å
α = 68.351 (3)°
β = 83.187 (4)°
γ = 89.367 (3)°
*V* = 582.01 (5) Å^3^
*Z* = 2
Mo *K*α radiation
μ = 0.28 mm^−1^
*T* = 294 K
0.16 × 0.15 × 0.08 mm

#### Data collection

Nonius KappaCCD diffractometer
Absorption correction: none
4433 measured reflections
2427 independent reflections
1753 reflections with *I* > 2σ(*I*)
*R*_int_ = 0.028

#### Refinement

*R*[*F* ^2^ > 2σ(*F* ^2^)] = 0.048
*wR*(*F* ^2^) = 0.135
*S* = 1.05
2427 reflections
154 parameters
H-atom parameters constrained
Δρ_max_ = 0.32 e Å^−3^
Δρ_min_ = −0.28 e Å^−3^

### 

Data collection: *COLLECT* (Enraf–Nonius, 2000 ▶); cell refinement: *SCALEPACK* (Otwinowski & Minor, 1997 ▶); data reduction: *DENZO* (Otwinowski & Minor, 1997 ▶) and *SCALEPACK*; program(s) used to solve structure: *SHELXS97* (Sheldrick, 2008 ▶); program(s) used to refine structure: *SHELXL97* (Sheldrick, 2008 ▶); molecular graphics: *ORTEP-3 for Windows* (Farrugia, 1997 ▶); software used to prepare material for publication: *WinGX* (Farrugia, 1999 ▶).

## Supplementary Material

Crystal structure: contains datablocks global, I. DOI: 10.1107/S1600536808015250/xu2426sup1.cif

Structure factors: contains datablocks I. DOI: 10.1107/S1600536808015250/xu2426Isup2.hkl

Additional supplementary materials:  crystallographic information; 3D view; checkCIF report

## Figures and Tables

**Table 1:** Hydrogen-bond geometry (Å, °)

*D*—H⋯*A*	*D*—H	H⋯*A*	*D*⋯*A*	*D*—H⋯*A*
N1—H1⋯O2	0.86	2.24	2.672 (3)	111
N2—H2⋯O1	0.86	2.00	2.677 (3)	135
N2—H2⋯O1^i^	0.86	2.43	3.091 (3)	133

Symmetry code: (i) 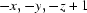.

**Fig. 1.**
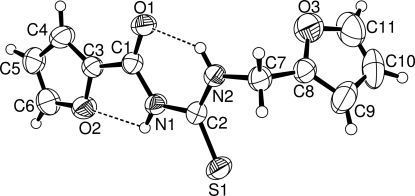
Molecular structure (50% probability displacement ellipsoids). Intramolecular hydrogen bonds are shown as dashed lines.

**Fig. 2.**
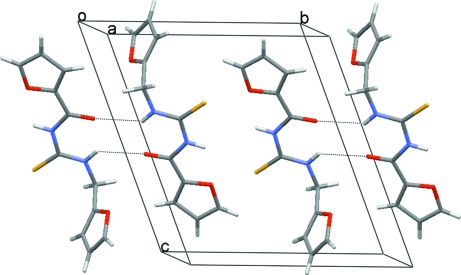
View of the crystal packing of the title compound. Intermolecular hydrogen bonds are shown as dashed lines.

## supplementary crystallographic information

### Comment

Thiourea and its derivatives have found extensive applications in the fields of medicine, agriculture and analytical chemistry. Thioureas are also widely used in heterocyclic syntheses (Dhooghe *et al.*, 2005). Aroylthioureas have also been found to have applications in metal complexes and molecular electronics (Aly *et al.*, 2007). The title compound (Fig. 1), has been successfully used as ionophore in amperometric sensors for Cd(II) (Estévez-Hernández *et al.*, 2007).

The title compound crystallizes in the thioamide form. The furoyl and furfuryl groups are *trans* and *cis*, respectively, to the S atom across the thiourea C—N bonds (Fig. 1). The main bond lengths and torsion angles are within the ranges obtained for similar compounds (Koch *et al.*, 2001). The C2—S1 and C1—O1 bonds show a typical double bond character with bond lengths (Table 1) of 1.661 (2) and 1.227 (2) Å respectively, closely related to other thiourea derivatives (Yamin & Hassan, 2004). However, all the C—N bonds (Table 1) of thiourea fragment C1—N1, C2—N1 and C2—N2 are in the range 1.392 (3)–1,327 (3) Å, intermediate between those expected for single and double C—N bonds (1.47 and 1.27 Å respectively). It is deduced that this thiourea moiety makes up a multi-electron conjugated π bond. The central thiourea fragment makes torsion angles of 2.5 (3)° and 88.1 (2)° with the furan carbonyl ring (O2-C3-C1-N1) and the furfuryl group (C2-N2-C7-C8), respectively. The *trans*-*cis* geometry in the thiourea moiety is stabilized by the N2—H2···O1 intramolecular hydrogen bond (Fig.1 and Table 2). An additional intramolecular hydrogen bond N1—H1···O2 is observed. In the crystal structure symmetry related molecules are linked by two N2—H2···O1 intermolecular hydrogen bonds to form dimers along the [010] direction (Fig. 2 and Table 2).

### Experimental

The title compound was synthesized according to a previous report (Otazo *et al.*, 2001), by converting furoyl chloride into furoyl isothiocyanate and then condensing with furfurylamine. The resulting solid product was crystallized from ethanol yielding X-ray quality single crystals (m.p 79–80 ° C). Elemental analysis (%) for C_11_H_10_N_2_O_3_S calculated: C 52.80, H 4.00, N 11.20, S 12.80; found: C 52.83, H 4.07, N 11.21, S 12.81.

### Refinement

H atoms were placed in calculated positions with N—H = 0.88 Å and C—H = 0.93 (aromatic) or 0.97 Å (methylene), and refined in riding model with U_iso_(H) = 1.2U_eq_(C,N).

### Crystal data

**Table:**

C_11_H_10_N_2_O_3_S	*Z* = 2
*M**_r_* = 250.27	*F*_000_ = 260
Triclinic, *P*1	*D*_x_ = 1.428 Mg m^−^^3^
Hall symbol: -P 1	Mo *K*α radiation λ = 0.71073 Å
*a* = 4.5999 (2) Å	Cell parameters from 2356 reflections
*b* = 11.3792 (6) Å	θ = 2.9–26.7º
*c* = 12.0556 (5) Å	µ = 0.28 mm^−^^1^
α = 68.351 (3)º	*T* = 294 K
β = 83.187 (4)º	Prism, colourless
γ = 89.367 (3)º	0.16 × 0.15 × 0.08 mm
*V* = 582.01 (5) Å^3^	

### Data collection

**Table:**

Nonius KappaCCD diffractometer	*R*_int_ = 0.028
ω scans	θ_max_ = 26.6º
Absorption correction: none	θ_min_ = 3.1º
4433 measured reflections	*h* = −5→5
2427 independent reflections	*k* = −14→14
1753 reflections with *I* > 2σ(*I*)	*l* = −15→13

### Refinement

**Table:**

Refinement on *F*^2^	H-atom parameters constrained
Least-squares matrix: full	*w* = 1/[σ^2^(*F*_o_^2^) + (0.0698*P*)^2^ + 0.1199*P*] where *P* = (*F*_o_^2^ + 2*F*_c_^2^)/3
*R*[*F*^2^ > 2σ(*F*^2^)] = 0.048	(Δ/σ)_max_ < 0.001
*wR*(*F*^2^) = 0.135	Δρ_max_ = 0.32 e Å^−^^3^
*S* = 1.05	Δρ_min_ = −0.28 e Å^−^^3^
2427 reflections	Extinction correction: none
154 parameters	

### Special details

**Table:**

Geometry. All e.s.d.'s (except the e.s.d. in the dihedral angle between two l.s. planes) are estimated using the full covariance matrix. The cell e.s.d.'s are taken into account individually in the estimation of e.s.d.'s in distances, angles and torsion angles; correlations between e.s.d.'s in cell parameters are only used when they are defined by crystal symmetry. An approximate (isotropic) treatment of cell e.s.d.'s is used for estimating e.s.d.'s involving l.s. planes.

### Fractional atomic coordinates and isotropic or equivalent isotropic displacement parameters (Å^2^)

**Table:**

	*x*	*y*	*z*	*U*_iso_*/*U*_eq_	
S1	−0.16463 (15)	0.45844 (5)	0.35901 (6)	0.0578 (2)	
O1	0.1719 (4)	0.07028 (14)	0.56626 (15)	0.0540 (4)	
O2	0.4479 (4)	0.28509 (15)	0.68097 (15)	0.0565 (4)	
N2	−0.1523 (4)	0.21274 (16)	0.39894 (15)	0.0417 (4)	
H2	−0.0911	0.139	0.4381	0.05*	
O3	0.0164 (5)	0.1731 (2)	0.16872 (17)	0.0793 (6)	
N1	0.1109 (4)	0.28290 (16)	0.51574 (15)	0.0421 (4)	
H1	0.1601	0.3461	0.5332	0.05*	
C8	−0.1860 (5)	0.2555 (2)	0.1856 (2)	0.0476 (5)	
C7	−0.3440 (5)	0.2252 (2)	0.30733 (19)	0.0461 (5)	
H7A	−0.4809	0.2911	0.3054	0.055*	
H7B	−0.4567	0.1465	0.3299	0.055*	
C3	0.3974 (5)	0.17197 (19)	0.67285 (18)	0.0415 (5)	
C1	0.2185 (5)	0.16912 (19)	0.58107 (18)	0.0411 (5)	
C2	−0.0679 (5)	0.30996 (19)	0.42464 (18)	0.0396 (5)	
C4	0.5302 (6)	0.0818 (2)	0.7549 (2)	0.0606 (7)	
H4	0.5303	−0.004	0.7676	0.073*	
C9	−0.2028 (7)	0.3500 (3)	0.0816 (3)	0.0771 (9)	
H9	−0.3219	0.4193	0.068	0.092*	
C6	0.6146 (6)	0.2632 (3)	0.7713 (2)	0.0611 (7)	
H6	0.6806	0.325	0.7966	0.073*	
C5	0.6702 (6)	0.1424 (3)	0.8184 (2)	0.0607 (7)	
H5	0.7805	0.1043	0.8814	0.073*	
C10	0.0005 (8)	0.3236 (4)	−0.0059 (3)	0.0850 (10)	
H10	0.0366	0.3725	−0.0875	0.102*	
C11	0.1238 (8)	0.2192 (4)	0.0502 (3)	0.0893 (10)	
H11	0.2657	0.1811	0.0144	0.107*	

### Atomic displacement parameters (Å^2^)

**Table:**

	*U*^11^	*U*^22^	*U*^33^	*U*^12^	*U*^13^	*U*^23^
S1	0.0793 (5)	0.0362 (3)	0.0594 (4)	0.0082 (3)	−0.0299 (3)	−0.0131 (3)
O1	0.0709 (11)	0.0387 (8)	0.0580 (10)	0.0093 (7)	−0.0252 (8)	−0.0195 (7)
O2	0.0688 (11)	0.0463 (9)	0.0608 (10)	0.0043 (8)	−0.0235 (8)	−0.0225 (8)
N2	0.0499 (10)	0.0363 (9)	0.0390 (9)	0.0019 (7)	−0.0114 (8)	−0.0122 (7)
O3	0.0938 (15)	0.0759 (13)	0.0589 (12)	0.0162 (11)	0.0080 (10)	−0.0197 (10)
N1	0.0512 (10)	0.0335 (8)	0.0426 (9)	0.0010 (7)	−0.0137 (8)	−0.0128 (7)
C8	0.0536 (13)	0.0444 (12)	0.0467 (12)	−0.0013 (10)	−0.0165 (10)	−0.0158 (10)
C7	0.0458 (12)	0.0479 (12)	0.0472 (12)	0.0008 (9)	−0.0131 (10)	−0.0185 (10)
C3	0.0437 (11)	0.0406 (11)	0.0420 (11)	0.0019 (9)	−0.0077 (9)	−0.0167 (9)
C1	0.0428 (11)	0.0400 (11)	0.0384 (11)	0.0012 (9)	−0.0034 (9)	−0.0125 (9)
C2	0.0413 (11)	0.0399 (11)	0.0350 (10)	0.0013 (9)	−0.0052 (8)	−0.0107 (9)
C4	0.0782 (17)	0.0469 (13)	0.0612 (15)	0.0147 (12)	−0.0309 (13)	−0.0189 (11)
C9	0.098 (2)	0.0648 (17)	0.0581 (17)	−0.0002 (16)	−0.0300 (16)	−0.0041 (14)
C6	0.0649 (16)	0.0689 (17)	0.0599 (15)	−0.0010 (13)	−0.0212 (13)	−0.0317 (13)
C5	0.0655 (16)	0.0704 (17)	0.0512 (14)	0.0123 (13)	−0.0272 (12)	−0.0229 (12)
C10	0.102 (2)	0.106 (3)	0.0390 (15)	−0.034 (2)	−0.0080 (15)	−0.0166 (16)
C11	0.108 (3)	0.098 (3)	0.0561 (19)	−0.010 (2)	0.0174 (18)	−0.0309 (19)

### Geometric parameters (Å, °)

**Table:**

S1—C2	1.661 (2)	C7—H7A	0.97
O1—C1	1.227 (2)	C7—H7B	0.97
O2—C3	1.351 (3)	C3—C4	1.336 (3)
O2—C6	1.353 (3)	C3—C1	1.465 (3)
N2—C2	1.327 (3)	C4—C5	1.412 (4)
N2—C7	1.460 (3)	C4—H4	0.93
N2—H2	0.86	C9—C10	1.439 (5)
O3—C11	1.359 (3)	C9—H9	0.93
O3—C8	1.366 (3)	C6—C5	1.314 (4)
N1—C1	1.367 (3)	C6—H6	0.93
N1—C2	1.392 (3)	C5—H5	0.93
N1—H1	0.86	C10—C11	1.294 (5)
C8—C9	1.327 (3)	C10—H10	0.93
C8—C7	1.476 (3)	C11—H11	0.93
			
C3—O2—C6	106.57 (19)	N1—C1—C3	115.07 (18)
C2—N2—C7	123.03 (18)	N2—C2—N1	116.40 (17)
C2—N2—H2	118.5	N2—C2—S1	125.21 (16)
C7—N2—H2	118.5	N1—C2—S1	118.38 (15)
C11—O3—C8	107.1 (2)	C3—C4—C5	106.4 (2)
C1—N1—C2	128.53 (18)	C3—C4—H4	126.8
C1—N1—H1	115.7	C5—C4—H4	126.8
C2—N1—H1	115.7	C8—C9—C10	106.1 (3)
C9—C8—O3	109.2 (2)	C8—C9—H9	126.9
C9—C8—C7	132.7 (3)	C10—C9—H9	126.9
O3—C8—C7	118.09 (19)	C5—C6—O2	110.6 (2)
N2—C7—C8	113.74 (18)	C5—C6—H6	124.7
N2—C7—H7A	108.8	O2—C6—H6	124.7
C8—C7—H7A	108.8	C6—C5—C4	106.7 (2)
N2—C7—H7B	108.8	C6—C5—H5	126.6
C8—C7—H7B	108.8	C4—C5—H5	126.6
H7A—C7—H7B	107.7	C11—C10—C9	107.2 (3)
C4—C3—O2	109.73 (19)	C11—C10—H10	126.4
C4—C3—C1	132.6 (2)	C9—C10—H10	126.4
O2—C3—C1	117.69 (18)	C10—C11—O3	110.3 (3)
O1—C1—N1	123.86 (19)	C10—C11—H11	124.8
O1—C1—C3	121.08 (19)	O3—C11—H11	124.8
			
C11—O3—C8—C9	−0.4 (3)	C7—N2—C2—S1	0.2 (3)
C11—O3—C8—C7	178.2 (2)	C1—N1—C2—N2	1.8 (3)
C2—N2—C7—C8	88.1 (2)	C1—N1—C2—S1	−179.47 (17)
C9—C8—C7—N2	−122.7 (3)	O2—C3—C4—C5	0.4 (3)
O3—C8—C7—N2	59.2 (3)	C1—C3—C4—C5	−179.9 (2)
C6—O2—C3—C4	−0.5 (3)	O3—C8—C9—C10	0.7 (3)
C6—O2—C3—C1	179.72 (19)	C7—C8—C9—C10	−177.6 (2)
C2—N1—C1—O1	0.9 (4)	C3—O2—C6—C5	0.4 (3)
C2—N1—C1—C3	−178.86 (19)	O2—C6—C5—C4	−0.2 (3)
C4—C3—C1—O1	−2.1 (4)	C3—C4—C5—C6	−0.1 (3)
O2—C3—C1—O1	177.7 (2)	C8—C9—C10—C11	−0.7 (3)
C4—C3—C1—N1	177.7 (2)	C9—C10—C11—O3	0.5 (4)
O2—C3—C1—N1	−2.5 (3)	C8—O3—C11—C10	−0.1 (4)
C7—N2—C2—N1	178.88 (18)		

### Hydrogen-bond geometry (Å, °)

**Table:**

*D*—H···*A*	*D*—H	H···*A*	*D*···*A*	*D*—H···*A*
N1—H1···O2	0.86	2.24	2.672 (3)	111
N2—H2···O1	0.86	2.00	2.677 (3)	135
N2—H2···O1^i^	0.86	2.43	3.091 (3)	133

Symmetry codes: (i) −*x*, −*y*, −*z*+1.
